# sPop: Age-structured discrete-time population dynamics model in C, Python, and R

**DOI:** 10.12688/f1000research.15824.3

**Published:** 2020-09-28

**Authors:** Kamil Erguler

**Affiliations:** 1 The Cyprus Institute, Climate and Atmosphere Research Center (CARE-C), 20 Konstantinou Kavafi Street, 2121, Aglantzia, Nicosia, Cyprus

**Keywords:** deterministic, stochastic, vector, population, model, age-specific, survival, development, dynamic, difference equations, C, Python, R

## Abstract

This article describes the sPop packages implementing the deterministic and stochastic versions of an age-structured discrete-time population dynamics model. The packages enable mechanistic modelling of a population by monitoring the age and development stage of each individual. Survival and development are included as the main effectors and they progress at a user-defined pace: follow a fixed rate, delay for a given time, or progress at an age-dependent manner. The model is implemented in C, Python, and R with a uniform design to ease usage and facilitate adoption. Early versions of the model were previously employed for investigating climate-driven population dynamics of the tiger mosquito and the chikungunya disease spread by this vector. The sPop packages presented in this article enable the use of the model in a range of applications extending from vector-borne diseases towards any age-structured population including plant and animal populations, microbial dynamics, host-pathogen interactions, infectious diseases, and other time-dependent epidemiological processes.

## Introduction

Heterogeneity is inherent in most naturally occurring populations. Individuals possess or attain in time certain characteristics, which could result in differences in behaviour or response to stimuli. Time-dependent heterogeneity may result in the stratification of a population into chronological units. This could introduce time delays to certain processes and might have a strong non-linear impact on dynamics ^1^.

For instance, distinct physiological stages emerge during insect development where each stage reacts differently to environmental factors such as temperature ^2^. In addition, the minimum incubation period of an infection requires time delays, which are often ignored in canonical modelling approaches ^3^. Time-dependent heterogeneity is ubiquitous in many areas including life sciences, engineering, and social sciences ^4^. The analysis of lifetime data ^4^, the degree day methodology ^5^, and age- or stage-structured population dynamics modelling ^6^ are common approaches for investigating such phenomena.

Incorporating age dependency in mathematical models can be challenging due to the need to keep track of the age of each individual in a population. A common work-around is to introduce predetermined intermittent stages to account for the different characteristics of each stage and the time it takes to pass from one to another. This approach has been extensively used in various contexts, *e.g.* to model animal development ^7^, insect life cycle ^8, 9^, disease transmission ^10, 11^, and economic surplus ^12^. Although intermittent stages are capable of representing age-structured populations to a certain extent, a large number of age classes are required for accuracy. Consequently, model development becomes a non-trivial task.

Numerous packages including popbio ^13^, demogR ^14^, and bayesPop ^15^ have been implemented in R to facilitate modelling and analysis of age- and stage-structured projection matrix models. As a viable alternative, Kettle and Nutter implemented an R package for agestructured population dynamics, StagePop, which offers true time delays in continuous time domain using deterministic delay differential equations ^16^.

Here, I present an alternative age-structured population dynamics model based on the population dynamics and disease-transmission models described in Erguler *et al.* 2016 ^17^ and 2017 ^18^. The approach involves automatically classifying a population into distinct age and development groups and applying a dynamic projection matrix to derive the next state. In its current form, the model is based on discrete-time difference equations. Three implementations of the model exists (the sPop packages) for three programming languages, C, Python, and R. The sPop packages provide a flexible number of age and development categories, include both deterministic and stochastic dynamics, and offer high-speed simulations to facilitate parameter inference.

The following section describes the theory behind the model and presents the use of each implementation with a commonly encountered case. The same case is modelled with each sPop package to emphasise the nuances in their usage. The Temporal resolution and accuracy section investigates the accuracy of numerical simulations with regards to different time step sizes. The Use cases section concludes with the sPop implementations of a short list of well-known mathematical models selected from a range of disciplines.

## Models and software

### The age-structured population dynamics (sPop) model

sPop is a discrete-time age-structured population dynamics model which comprises mainly of the survival and development processes. While ageing, pertinent to survival, is the dominant process, development is merely a label assigned to the individual as a merit of surviving long enough to enable physiological maturation. As expected, development takes place as long as survival is assured.

The earliest version of the sPop model ^17^ followed the degree day approach and represented development as an accumulative process; a unit of progress accumulated at each iteration until a threshold was reached. The rate of accumulation could be fixed or variable in response to external or internal factors. When the value of this indicator exceeded a predefined threshold, the process of development (or survival) was considered complete. The more recent version of the model ^18^ employed a hazard function, and observed the probability of the indicator exceeding the threshold at each iteration. Following an unsuccessful outcome, *i.e.* survival, a unit of progress corresponding to one time step was accumulated. The exchange facilitated the development of both deterministic and stochastic models with uncertainty in stage durations or lifespan, and thus is employed for the current implementation of the sPop packages.

In this context, age represents the number of iterations an individual spends alive from birth, and development is the number of iterations spent in a particular stage. The duration of a development stage — the temporal boundary between successive stages — can be defined as needed. If absolute survival is assured and two-stage development is assumed — no alternative stages to develop into — then the development process resembles the survival process. In other words, we can conceptualise the states of being alive and dead as analogous to the two stages of development. Following the line of thought, we take the survival process as the basis of discussion, and note that applicability extends to the development process as long as survival is assured.

The prevailing assumption of the discrete time process is that the survival (or development) probability, although allowed to vary in the long term, does not significantly change during the time interval of a single iteration. In addition, we assume that individuals at the same age and degree of development behave identically and can be grouped together. As a result, death can be considered as a binomial process both at the individual and population levels; the number of steps taken alive follows a geometric distribution while the number of individuals surviving a single step follows a binomial distribution.

If a population is composed of a cohort of x individuals and each has a probability *pX _τ_* of dying during iteration τ, the number of individuals in the subsequent time interval can be written as

*x _τ_*_+1_ ∼ *Binom(x _τ_,*1− *pX _τ_*).                                   (1)

The expected number of individuals in subsequent iterations can be written accordingly.

$$\begin{array}{l} \text{〈}x_{1}\text{〉}=\left(1-pX_{0}\right)x_{0}\\ \text{〈}x_{2}\text{〉}=\sum_{x_{2}=0}^{x_{0}}x_{2}\;\sum_{x_{1}=x_{2}}^{x_{0}}\text{Φ}\left(x_{0},\;1-pX_{0},x_{1}\right)\;\text{Φ}\left(x_{1},\;1-pX_{1},x_{2}\right)\;\\ \;=\left(1-pX_{0}\right)\left(1-pX_{1}\right)x_{0}\\ \;\vdots \end{array}$$

where Φ(n, p, k) is the probability mass function of the binomial distribution with size n and probability p at k. Consequently, the expected behaviour of Equation 1 is

*x _τ_*_+1_ = (1− *pX*_τ_) *x _τ_*,                                            (2)

which is employed for deterministic simulations. It is worth noting that the deterministic sPop model resembles the Leslie age-structured population dynamics model with 1 − *pX _τ_* as the survival fraction per age class ^19^.

For certain natural species, death can be considered as a spontaneous event with a fixed probability per unit time; yet, for others, mortality may increase with the age of an individual. The sPop model allows age-structured population dynamics to attain various forms depending on the chosen probability regime.

**Fixed duration:** Survival with a fixed lifespan entails a stepwise transition to death following a given number of iterations. The probability of dying during iteration τ can thus be written using the step function

$$pX_{\tau }=\{\begin{array}{cc} 0 & \tau <d\\ 1 & \tau \geq d \end{array}\;,$$

where d is the lifespan of an individual in terms of the number of iterations.

**Fixed probability:** If the probability of death is assumed constant and time invariant, *pX*, the number of iterations to its first (and clearly last) occurrence can be described with a geometric distribution, Geom(1− *pX*). Similarly, in continuous time, a fixed probability of death per time unit leads to an exponentially distributed time of death, Exp(1 − *pX*).

The dynamics of a population with a fixed survival probability can be described with Equation 1 in the stochastic and Equation 2 in the deterministic scenario by setting *pX _τ_* = *pX*.

The fixed-probability scenario can be associated with the canonical age-independent dynamical modelling approaches: the master equation ^20^ and the ordinary differential equation (ODE). In the case of an infinitesimally small time step, the probability of multiple events taking place in a single iteration approaches zero. Consequently, the time evolution of the state of the population can be described with a continuous-time master equation for spontaneous decay,

$$\frac{d}{dt}\mathrm{Pr}(x;t)=pX(x+1)\mathrm{Pr}(x+1;t)-p_{X}x\mathrm{Pr}(x;t),$$

where *pXx* is the probability of a death event taking place per unit time in a population of x individuals ^20^. Please note that we employ t to refer to time while reserving τ for the number of iterations.

As population size increases, the intrinsic stochastic variability decreases, and the emerging dynamics can be described with the ODE

$$\frac{d}{dt}x=-pXx,$$

where *pX* represents the rate of death per individual per unit time. The ODE can be considered as analogous to the deterministic difference equation in Equation 2.

In order to construct an age-dependent model, in addition to allowing *pX* to change in time, the heterogeneity in a population of individuals at different ages should be recorded. The latter can be achieved by using a continuous density function or an array of discretised age classes. The sPop packages incorporate a dynamic array of discrete age and development classes, which can be used to represent the two processes in a fine temporal resolution limited only by the availability of computational resources.

In age-dependent survival, with age indicating the degree of completeness of the survival process, the probability of dying during iteration τ depends on the probability of surviving until τ. Consequently, *pX _τ_* can be written in terms of the hazard function,

$$pX_{\tau }=\{\begin{array}{ll} \frac{f(\tau +1)-f(\tau )}{1-f(\tau )} & f(\tau )\neq 1\\ 1 & f(\tau )=1 \end{array}\;,\;(3)$$

where f(τ) is the cumulative probability of death until the beginning of iteration τ.

Age-dependent mortality, f(τ), can attain various forms depending on the underlying biological ageing process. Gamma and negative binomial distributions are included in the sPop packages to represent survival (and development) in continuous and discrete time settings, respectively.

**Gamma-distributed lifetime:** Gamma distribution is commonly used to represent age-dependent survival as part of parametric survival analysis ^21^. It is the generalised form of the Erlang distribution describing the sum of independent exponentially distributed random numbers ^22^.

We discretise the continuous-time gamma distribution to derive a first order approximation for the probability of dying during an iteration. We define f(τ) in Equation 3 as

$$f(\tau )=\frac{\gamma (k,\tau /\theta )}{\Gamma (k)},$$

where

$$k\;=\;\frac{\mu^{2}}{\sigma^{2}}\;\mathrm{and}\;\theta \;=\;\frac{\sigma^{2}}{\mu }.$$

In this equation, γ(a,x) is the lower incomplete gamma function, Γ(a) is the complete gamma function, μ is the expected lifespan, and σ is the standard deviation of lifespan in terms of the number of iterations.

**Negative binomial-distributed lifetime:** Negative binomial distribution represents the distribution of the sum of n independent geometrically distributed random numbers. As an alternative representation of the survival process in discrete time, we employ the negative binomial distribution by defining f(τ) in Equation 3 as

$$f\left(\tau \right)\;=\;\frac{B\left(p;r,\tau +1\right)}{B\left(r,\tau +1\right)},$$

where B(r, τ + 1) and B(p; r, τ + 1) are the complete and incomplete beta functions, respectively, and,

$$p=\frac{\mu }{\sigma^{2}}\;\text{and}\;r=\frac{\mu^{2}}{\sigma^{2}-\mu }\;\forall \sigma^{2}>\mu .$$

As in the case of gamma-distributed lifespan, μ represents the average and σ represents the standard deviation of lifespan in terms of the number of iterations. Despite its compatibility with the discrete time steps of the sPop model, the negative binomial distribution is restrictive over the minimum allowed standard deviation for a given mean. Although it describes a collection of an integer number of events, r, its definition extends to the positive real domain, the specific form which is known as a Polya distribution ^23^.

### The sPop packages

The sPop packages help to incorporate age-structured populations in discrete-time deterministic and stochastic difference equations models in C, Python, and R. The packages dynamically stratify a population to follow individuals through successive time steps. Survival and development may (i) progress at a fixed propensity, (ii) delay for a given number of iterations, (iii) follow a gamma-distributed (or negative binomial-distributed) lifetime, (iv) halt for a given time period, or (v) follow any user-defined scheme.

In this context, propensity is defined as the probability of an individual (the stochastic version) or the fraction of a population (the deterministic version) dying or developing per unit time. Age (t _α_) and development (t _δ_) are the two counters of elapsed time. Age is pertinent to survival and development marks the time invested in the development process. Each individual is allowed to stay in the population given that neither death nor development occurs during an iteration. Upon completion of development, a third counter, development cycle (t _π_), is incremented, t _δ_ is reset to zero, and the group of individuals is removed from the population. When modelling periodic development processes, such as the gonotrophic cycle or human pregnancy, or development with multiple stages, the group can be reintroduced to the population for the subsequent cycle or stage of development.

Human pregnancy can be given as an analogy; a woman’s age would be t _α_ and the stage of her pregnancy would be t _δ_. The population would then be comprised of a group of women in different stages of pregnancy. It is important to note that, since age and development progress in synchrony, their time units need to be the same, *e.g.* months or days. In this analogy, birth would be triggered by t _δ_, which would result in t _π_ being iterated, which could be seen as the number of births each mother has given.

## Implementation

The R implementation of sPop is available on CRAN as the albopictus package (v.0.5) and on the GitHub repository https://doi.org/10.5281/zenodo.1685054. The C and Python implementations are available as part of the albopictus package (v.1.11.0) on PyPI and the GitHub repository https://doi.org/10.5281/zenodo.1685289. The packages are implemented for R version 3.5.1 and Python version 3.7.0.

This section is reserved for outlining the use of each implementation to model the same theoretical population where both development and survival are age-dependent and gamma-distributed. In addition, the population exhibits a periodic development process with a mean duration of 50 hours and a standard deviation of 10 hours, and survival is a function of the number of development cycles,

$$\begin{array}{cc} \begin{array}{l} \mu =\max(240,480-48\;t_{\pi })\;\text{hours}\\ \sigma =\mu /10. \end{array} & \;(4) \end{array}$$

### R

Before we begin modelling, we load the albopictus package in R, and define the survival function as described in Equation 4.


R > library(albopictus)
R >
                            
R > death <- function(pop) {
R +     if (nrow(pop)==0)
R +         return(data.frame(mean=480, sd=48.0))
R +     mn <- 480.0 - (48.0 * pop$devcycle)
R +     mn[mn < 240.0] <- 240.0
R +     return(data.frame(mean=mn, sd=0.1*mn))
R + }


The function returns a data.frame with a desired mean and standard deviation. Next, we initiate a population by calling the initiation routine of the spop class.


R > vec <- spop(stochastic=TRUE, prob="gamma")


With this line, we construct a stochastic population model with the gamma distribution as the basis of survival and development. Setting prob to nbinom selects the negative binomial distribution instead.

In order to introduce the first batch of individuals, we use the add method.


R > add(vec) <- data.frame(number=1000)


By default, age, development cycle, and the duration of development will be set to zero for all individuals. These can be customised by supplying additional fields to the data.frame: age to set age, devcycle to set the number of development cycles, and development to set the number of iterations the current development cycle has taken.

We can directly access the population structure of the spop class to inspect the number of individuals grouped with respect to age, development cycle, and the degree of development. Here, we will use these information to calculate the mean and standard deviation of expected lifetime for each age-development group.


R > tmp <- death(vec@pop)


The following step iterates the population for one time-unit by using the iterate method.


R > iterate(vec) <- data.frame(dev_mean = 50,
R +                            dev_sd = 10,
R +                            death_mean = tmp$mean,
R +                            death_sd = tmp$sd)


By defining dev_mean and dev_sd, we opt to use the gamma distribution to describe the probability of development. Setting dev_sd to zero results in the gamma distribution being discarded and a fixed number of iterations (indicated by dev_mean) being assigned for development. Instead, setting dev instead of dev_mean and dev_sd results in a daily constant development probability. The same principles apply for the survival process, where we provide the mean and the standard deviation of the gamma-distribution for each age- development group as calculated by the death function. After each iteration, the age and degree of development of the population are updated and the total number of individuals completing development is recorded together with the detailed account of the corresponding age-development groups. We access these data using the developed and devtable methods, respectively. In addition, the dead method returns the number of dead individuals following an iteration.


R > d <- developed(vec)
R > add(vec) <- devtable(vec)


In this example, we assume a periodic development process; therefore, we introduce all the individuals completing development back to the population using the add method.

Finally, we read the total size of the population using the size method.


R > s <- size(vec)


In the R implementation, we also provide an accessory method, perturb, to perform the same functions as the iterate method without updating age or development. By using perturb, the structure and size of a population can be modified to model the impact of an intervention or migration. The effect is immediate and in addition to the continuing survival and development processes. For instance, perturb can be used to model population control. If a certain agent kills a fraction of a population upon delivery, perturb can be used by specifying death to remove the affected individuals from the population. Otherwise, if the effect is age-dependent, death.mean and death.sd can be specified to calculate the fraction to be removed. If there is a need to migrate a subset of a population (population A) to a different population (population B), perturb can be used by specifying an appropriate development process ( dev for age-independent, dev.mean and dev.sd for age-dependent migration). This results in keeping a detailed record of the age and stage of development of the individuals removed from population A. Essentially, this is the list of individuals selected for migration, and it can be used as desired. A simple example of migration is given below.



R > A <- spop(stochastic=FALSE, prob="gamma")
R > B <- spop(stochastic=FALSE, prob="gamma")
R > add(A) <- data.frame(number=1000)
R > perturb(A) <- data.frame(dev=0.5, death=0)
R > add(B) <- devtable(A)



The above code results in the migration of 500 individuals from population A to B while freezing the age, development cycle, and development counters.

### Python

The Python implementation of the population dynamics model can be imported from the albopictus package.


Python >>> from albopictus.population import spop


We begin by declaring the survival function as in Equation 1.


Python >>> def death(pop):
Python ...     if pop.shape[1]==0:
Python ...         return [480.0, 48.0]
Python ...     mn = 480.0 - 48.0 * pop[:,1]
Python ...     mn[mn < 240.0] = 240.0
Python ...     return [mn, 0.1*mn]


Unlike the R implementation, the population structure is stored in a numpy.ndarray with the following order of columns: age, development cycle, degree of development, and number. Although the initiation step is similar to the R implementation, a two-dimensional list or a numpy.ndarray should be supplied to intriduce batches of individuals to a population.


Python >>> vec = spop(stochastic=True,prob="gamma")
Python >>> vec.add( [ [0, 0, 0, 1000] ] )


By using the add method above, we introduce 1000 individuals with zero age, development cycle, and degree of development. The population structure is directly accessible, which enables us to calculate a different mean and standard deviation for the gamma-distributed development of each age-development group.


Python >>> tmp = death(vec.pop)


Next, we iterate the population for one time-unit using the iterate method.


Python >>> vec.iterate(dev_mean = 50,
Python ...             dev_sd = 10,
Python ...             death_mean = tmp[0],
Python ...             death_sd = tmp[1])


In order to read the total number of individuals completing development, the detailed account of the corresponding age-development groups, and the total size of the population, we access the developed, devtable, and size attributes of the spop class. Please note that these attributes are overwritten each time the iterate method is called. Here, we record the total number of developed individuals and the population size, and reintroduce the developed individuals to the population for the next round of development.


Python >>> d = vec.developed
Python >>> vec.add(vec.devtable)
Python >>> s = vec.size


The Python implementation of the iterate method accepts an additional logical indicator pause to prevent updating age and development. If this parameter is supplied and if it is false, the iterate method acts as the perturb method of the R implementation.

### C

The C implementation of the sPop package is further optimised for speed. The source code resides in the albopictus package of Python, and it needs to be compiled with the GNU Scientific Library (version 2.1 or later). We begin by locating the package directory and compiling three source files into the object code. Assuming that the file name of our model is test_spop.c, we produce the executable with the following.


$ gcc -c -o ran_gen.o ${pkgdir}/ran_gen.c
$ gcc -c -o gamma.o ${pkgdir}/gamma.c
$ gcc -c -o spop.o ${pkgdir}/spop.c
$ gcc -I${pkgdir} -lgsl -o test_spop ran_gen.o
    gamma.o spop.o test_spop.c


where $pkgdir is a bash variable holding the package directory. In order to use the package, we need to include the following header files in test_spop.c.


C   #include "ran_gen.h"
C   #include "gamma.h"
C   #include "spop.h"


The first header file defines the routines required for random number generation, and the second one defines the routines for the gamma and negative binomial distributions. The last header file defines the spop population structure and the associated functions for initialisation, modification, and garbage collection.

Each age-development group is stored in the individual_st data structure,


C   typedef struct individual_st {
C     unsigned int age;
C     unsigned int devcycle;
C     unsigned int development;
C     sdnum number;
C   } individual_data;


where the age, development cycle, degree of development, and the number of individuals in each age-development group are stored in age, devcycle, development, and number variables in the same order. The sdnum is a union data structure holding an unsigned int for a stochastic population or a double for a deterministic population. The spop data structure holds an array of individuals ( individuals), population size ( size), the number of dead and developed individuals following an iteration ( dead and developed, respectively), a detailed account of developed individuals ( devtable), an indicator for the probability distribution of age dependence ( gamma_mode), a logical indicator for a stochastic or a deterministic model ( stochastic), and two counters to manage the dynamic size of individuals ( ncat and cat).


C   typedef struct population_st {
C     individual_data *individuals;
C     sdnum size;
C     sdnum dead;
C     sdnum developed;
C     void *devtable;
C     unsigned char gamma_mode;
C     unsigned char stochastic;
C     unsigned int ncat;
C     unsigned int cat;
C   } *spop;


Following the procedure in previous sections, we begin implementing the model in test_spop.c by declaring the survival function in Equation 1.


C   void death(const individual_data *ind,
C              double *death_prob,
C              double *death_mean,
C              double *death_sd) {
C     (*death_prob) = 0;
C     (*death_mean) = 480.0 - (ind->devcycle > 4 ?
     240.0 : 48.0 * ind->devcycle);
C     (*death_sd) = 0.1 * (*death_mean);
C   }


Please note that the C implementation handles a single age-development group at a time; therefore, the survival function is redesigned accordingly.

Next, we initiate a stochastic model with the gamma distribution as the basis of survival and development using the spop_init function with the first parameter set to a logical true.


C   vec = spop_init(1,MODE_GAMMA_HASH);


The macro MODE_GAMMA_HASH refers to the optimised implementation of the gamma distribution. Alternatively, MODE_NBINOM_RAW and MODE_GAMMA_RAW refer to the unoptimised implementations of the negative binomial and the gamma distributions. Optimisation involves recording previously-used values in a hash table for reuse, however, is memory intensive and should be used with caution. Faster more efficient implementations of the probability distributions are the main concern for future releases.

Having initiated vec, we introduce 1000 individuals of zero age with the spop_add function.


C   spop_add(vec,0,0,0,1000);


spop_add accepts parameters in the following order:

1. spop s: the spop data structure
2. unsigned int age: the age of individuals
3. unsigned int devcycle: the number of development cycles passed
4. unsigned int development: the degree of development of individuals
5. sdnum number: the number of individuals ( unsigned int or double)

In order to iterate the population for one time interval, we use the spop_iterate function.


C   spop_iterate(vec,
C                0,
C                50.0, 10.0,
C                0,
C                0,
C                0, 0,
C                death,
C                0);


spop_iterate accepts the following parameters in the given order:

1. spop s: the spop data structure
2. double dev_prob: fixed daily development probability (priority over the other development-related parameters)
3. double dev_mean: mean development time (gamma or negative binomial)
4. double dev_sd: standard deviation of the development time
5. iter_func dev_fun: development function (similar to the death function above)
6. double death_prob: fixed daily death probability (priority over the other survival-related parameters)
7. double death_mean: mean time of death (gamma or negative binomial)
8. double death_sd: standard deviation of the time of death
9. iter_func death_fun: survival function
10. unsigned char pause: logical indicator to prevent updating age and development

Following each iteration, the list of age-development groups that completed their development is stored in the devtable variable of the spop data structure. In order to reintroduce these individuals back to the population, we use the spop_popadd function.


C   spop_popadd(vec,vec->devtable);


It is possible to obtain a summary output of the population structure by using the spop_print function, which takes the spop data structure as the only parameter. spop can be recycled by emptying its contents with the spop_empty function.


C   spop_empty(vec);


Finally, in order to clear the memory used by vec, we supply its address to the spop_destroy function.


C   spop_destroy(&vec);


### Model output

**Figure 1.**
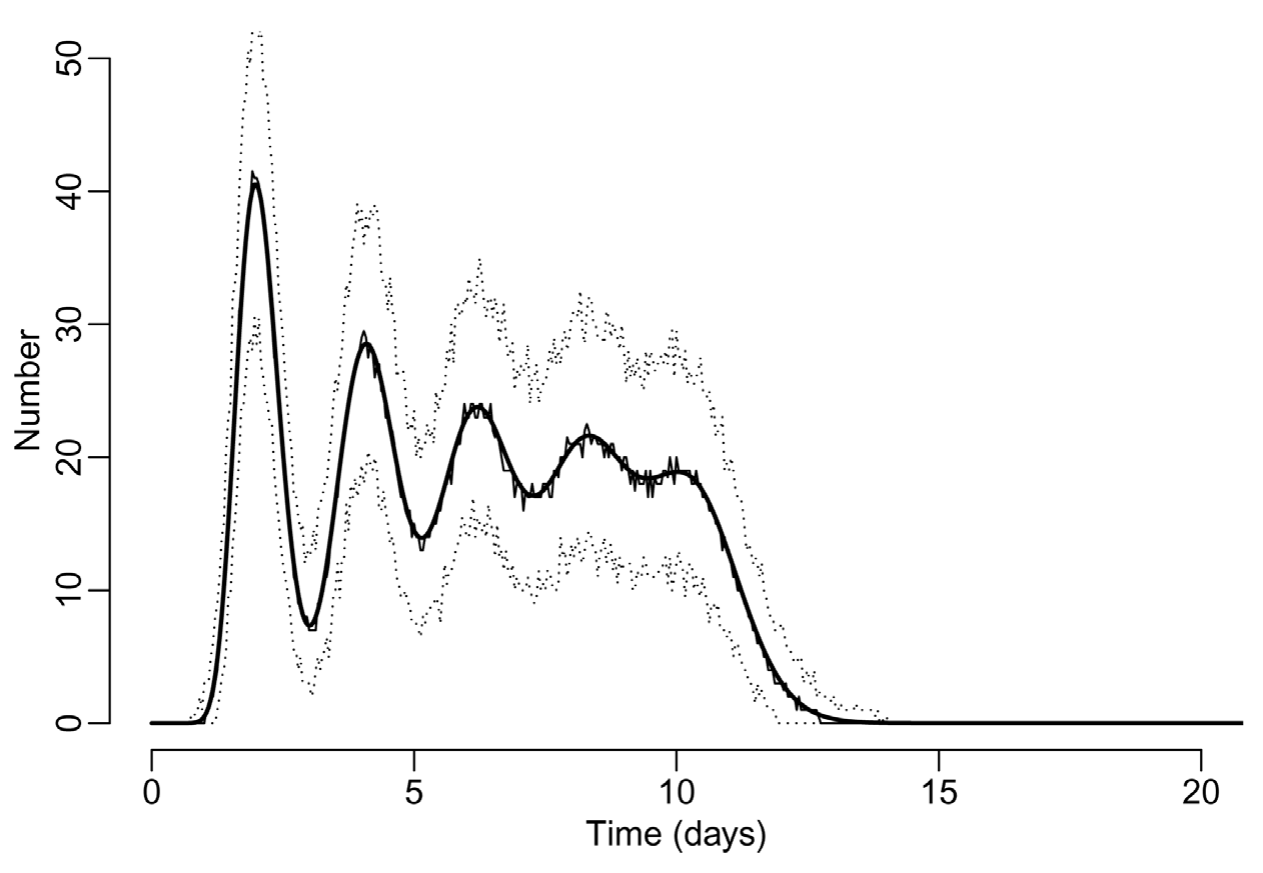
Number of individuals completing a development cycle in 20 days. Thick solid line indicates the mean trajectory from the deterministic simulation, while the thin solid line and the dotted lines indicate the median and the 95% range of the stochastic simulation output. The timestep for each iteration is one hour.

All three implementations of the model are given in the Extended Data ( test_spop.R, test_spop.py, and test_spop.c) ^24– 26^. The resulting distribution of the number of individuals completing a development cycle during the first 20 days of simulation is given in Figure 1.

Five cycles of development are clearly seen from the figure, while the population survives for less than 15 days with the survival function defined in Equation 4. Blending of development cycles is apparent and progressive due to the uncertainty in the duration of development (50 hours on average with a standard deviation of 10 hours).

## Temporal resolution and accuracy

In the previous section, we simulated a hypothetical age-structured population using 1-hour time steps. Here, we investigate the effect of time step size to the accuracy of simulations by using a simplified version of this model. We focus on the deterministic version and assume that the default development duration is 10 ( *±*2) days and the lifetime is 50 ( *±*10) days. We define a scaling factor *α* to tune step size between days and hours. The resulting Python code for the iteration of this population (named vec) is given below.


Python >>> vec.iterate(dev_mean = 10* alpha,
Python ...             dev_sd = 2*alpha,
Python ...             death_mean = 50*alpha,
Python ...             death_sd = 10*alpha)


In addition, we let *α* scale the number of iterations, which is by default 50. Consequently, when *α* = 1, the average lifetime is 50 and the simulation runs for 50 iterations each corresponding to 1 day. When *α* = 24, the average lifetime becomes 1200 and the number of iterations also becomes 1200, which implies that each iteration corresponds to 1 hour. It is straightforward to have intermediary time steps. For instance, *α* = 2 and *α* = 4 yield half-day and quarter-day iterations, respectively.

**Figure 2.**
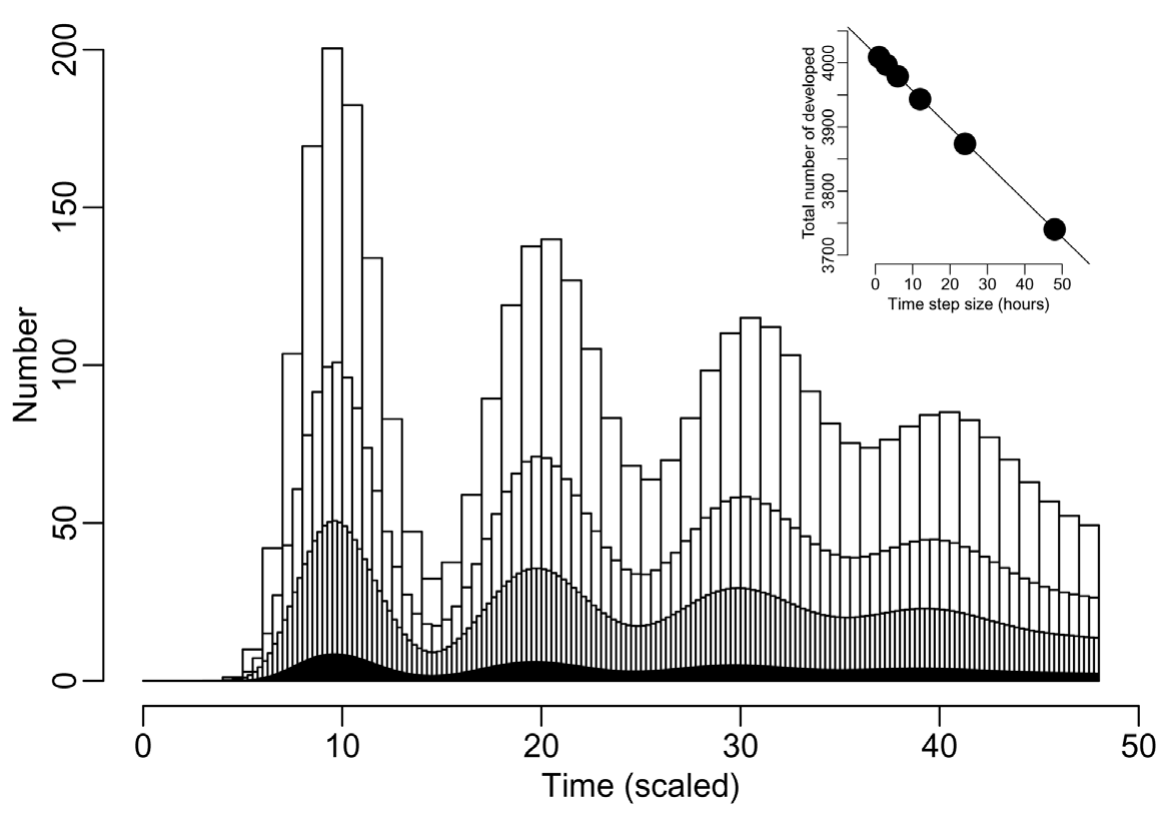
The effect of time step size to simulation accuracy. The number of individuals completing a development cycle is shown for different time steps. The widths of the bars correspond to the following time step sizes: 24 hours ( *α* = 1), 12 hours ( *α* = 2), 6 hours ( *α* = 4), and 1 hour ( *α* = 24) from the widest to the thinnest. The inset graph shows the total number of developed individuals with respect to different step sizes (solid dots). The line of best fit is also shown in the graph.

We demonstrate the effect of four time step sizes on the number of individuals completing a development cycle in Figure 2. The model yields oscillations fading in amplitude similar to the original model ( Figure 1). Although the peak height decreases, the cumulative number of developed individuals agree well in each case. In the inset of Figure 2, we show that the overall numerical error increases linearly with increasing time step size.

## Use cases

This section describes how the Python implementation of sPop can be used to model some of the well-known population dynamics models. These models can also be constructed in R and C by following the guidelines presented in the previous section.

### Nicholson’s blowflies

We begin with Nicholson’s Blowflies, a classic example of time-delayed stage-structured population model ^16, 27^. The model comprises five distinct life-stages and exhibits stable quasi-cyclic oscillations. Although, originally the model was constructed using continuous time-delay equations, we will demonstrate that the sPop model adheres well to the observed dynamics and the implementation presented in the StagePop package ^16^. Furthermore, we will present a stochastic version of the model, which helps to improve our understanding of the observed variation.

Both the deterministic and stochastic versions of the model are presented in the Extended data ( case_studies.py) ^28^. The adaptation assumes fixed daily survival and strict development durations, values of which are the same as the original model (Figure 2 in Gurney *et al.* 1983 ^27^). A scaling factor is introduced to calculate hourly instead of daily propensities to improve accuracy on a par with the continuous-time simulations.

**Figure 3.**
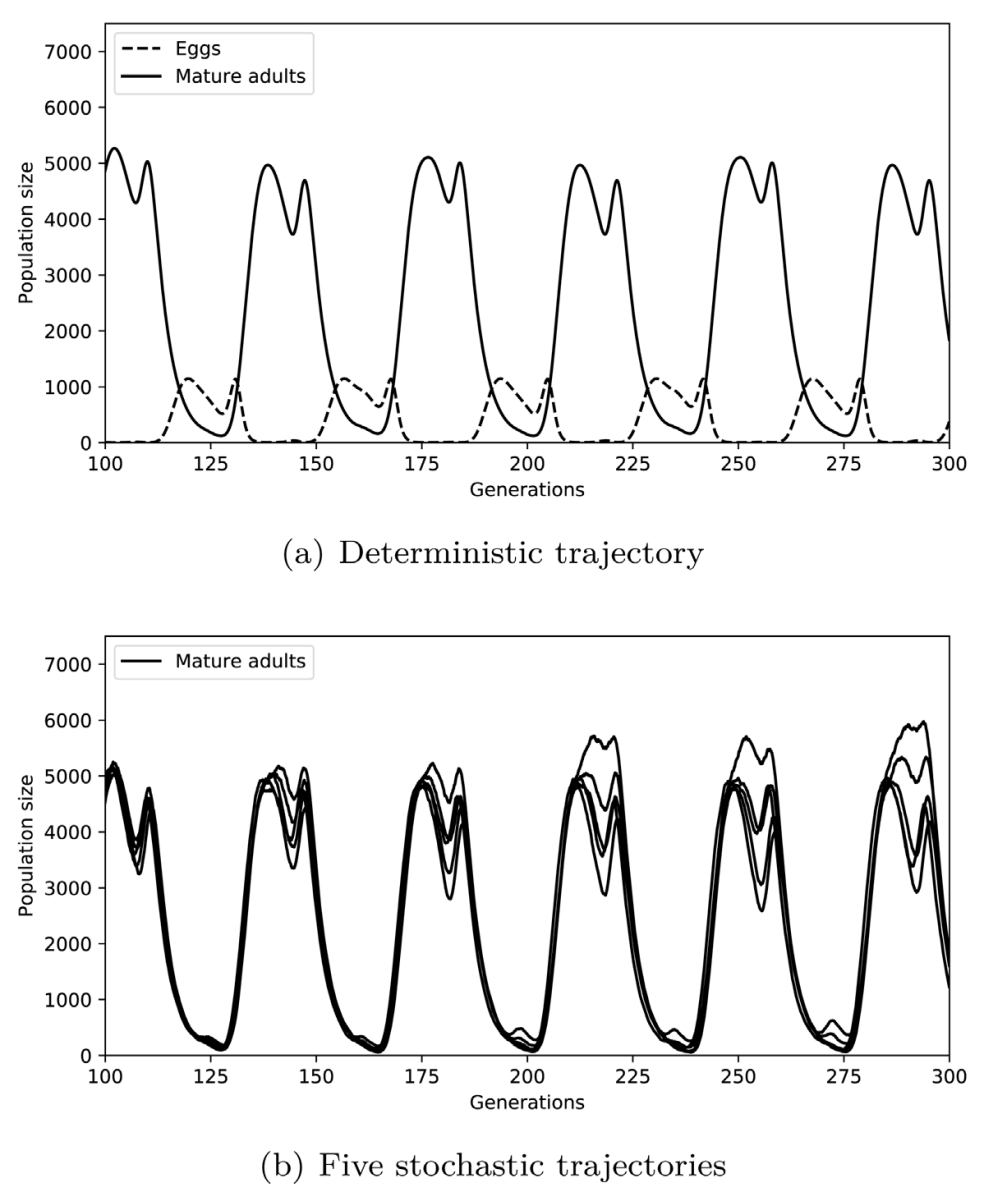
Age- and stage-structured model of Nicholson’s Blowflies in discrete time. In ( **a**), the deterministic trajectories of eggs (dashed line) and mature adults (solid line) are shown for the corresponding generations. In ( **b**), five stochastic trajectories of mature adults are shown for the same duration.

As a result, the output of the model is almost identical to the output of the original model (compare Figure 3(a) with the Figure 3a of Gurney *et al.* 1983 ^27^). The six peaks shown between generations 100 and 300 are matched by the stochastic version of the model ( Figure3(b)). Evidently, the highest variability in stochastic simulations is in peak amplitude, whereas the frequency of the oscillations is well conserved. During each peak, two sub-peaks are observed, which are separated by a trough. The heights of each peak and trough largely vary suggesting that the two peaks may not be resolved in the observations of natural populations. Nevertheless, similar fluctuations were observed in the laboratory culture of the Australian sheep blowfly reported in Nicholson 1954 ^29^ (also presented in Gurney *et al.* 1983 ^27^ Figure 1).

### Age-structured host-parasite interactions

Another classic example of age dependency in population dynamics was proposed by Hastings (1984) ^30^ as a variation of the host-parasite interaction model of Nicholson and Bailey (1935) ^31^. The Nicholson-Bailey model considers dynamics in discrete generations where parasites traverse a given area in search of a host. As a result, the number of parasites in the subsequent generation corresponds to the number of hosts parasitised. Hastings used this model for prey-predator interactions where he represented preys as hosts and predators as parasites. He introduced age-structure to the prey population and assumed that only juvenile preys are targeted by predators. Following Hastings, we use the terms host and parasite as analogous to prey and predator, respectively, regarding the emerging dynamics.

**Figure 4.**
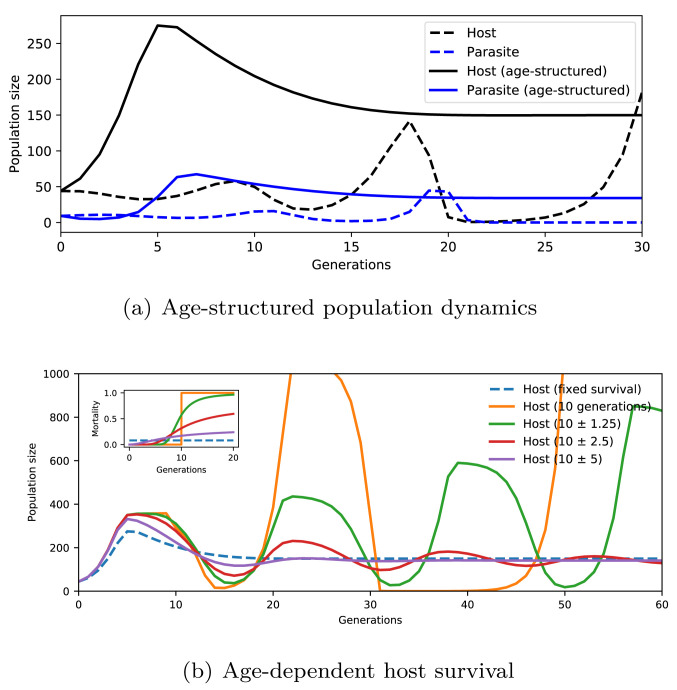
Age-structured host-parasite interactions of Nicholson-Bailey-Hastings. In ( **a**), the Nicholson-Bailey model (dashed lines) is compared with its age-structured version (solid lines) where parasites choose host in an age-dependent manner. The number of parasites is scaled down to 25% to aid visualisation. In ( **b**), the effect of age-dependent host survival on stability is shown. Mortality rate is given in the inset with respect to age (the number of generations). Dashed line: age-independent mortality; solid orange: life expectancy of precisely 10 generations; solid green, red, and purple: gamma-distributed life expectancy with mean 10 and standard deviation 1.25, 2.5, and 5 generations, respectively.

Both the original Nicholson-Bailey model and its age-structured version are implemented in the Extended data ( case_studies.py) ^32^. As shown in Figure 4(a), the original model without age dependency (dashed lines) exhibits oscillations with increasing amplitude around an unstable steady state. The model output matches Figure 10 in Nicholson and Bailey (1935) ^31^. Introducing age-structure with a fixed survival rate for host results in the stabilisation of dynamics as reported by Hastings (1984) ^30^ and seen in Figure 4(a) (solid lines).

Hastings (1984) ^30^ also discusses the disruptive effect of age-dependent host survival in stability. This is evident in Figure 4(b), where the survival imbalance between young and old individuals drives the dynamics away from the steady state, eventually rendering it unstable. When the age-dependent mortality curve is close to being horizontal, the dynamics closely resemble the Hastings’ model with no age limit (solid lines in Figure 4(a) and the dashed line in Figure 4(b)). When mortality is considerably higher in older individuals (green line in Figure 4(b)) or a strict age limit is introduced (orange line in Figure 4(b)), the stability is lost and the amplitude of oscillations increase in time.

### The Great Plague in Eyam

The final case we study is the severe outbreak of bubonic plague in Eyam (Sheffield, UK) in 1665–1666 ^32^. The outbreak was initially modelled by Raggett *et al.* 1982 ^32^, and later, a simple deterministic susceptible-infectious-removed (SIR) epidemic model was developed by Brauer *et al.* 2010 ^33^ to study the outbreak.

Although canonical SIR models are not age-structured, the underlying transmission dynamics is intricately time-dependent. This becomes clear when, for instance, the (intrinsic) incubation period is observed to be strictly more than a couple of days but not less. While a system of ordinary differential (or difference) equations describes an uninterrupted flow between the incubation period and the infectious state, the sPop age-structured model is able to track the length of the incubation period for each individual and trigger state change only for the right individuals at the right times.

**Figure 5.**
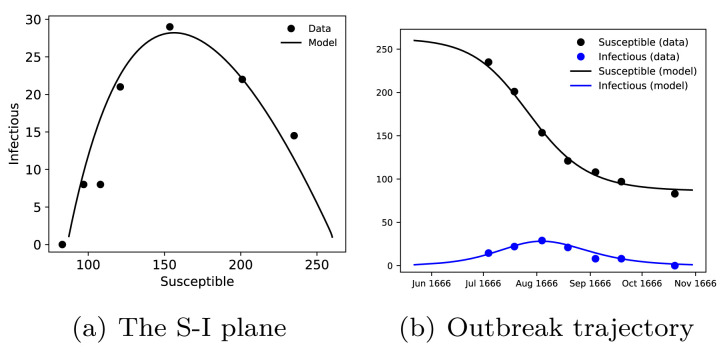
The age-structured SIR model of the Great Plague in Eyam. In ( **a**), the simulated numbers of infectious versus susceptible individuals are shown together with the outbreak data. In ( **b**), the number of susceptibles and infectious cases are plotted with respect to time for the duration of the outbreak. Model simulations (solid lines) are compared with the outbreak data (dots).

An age-structured version of the SIR epidemic model where the infectious stage duration is modelled with a gamma distribution is presented in the Extended Data ( case_studies.py) ^28^. As shown in Figure 5, the number of infectious cases with respect to the number of susceptible individuals, the S-I plane, ( Figure 5(a)) and the time trajectory of the outbreak ( Figure 5(b)) closely follow the data presented in Brauer *et al.* 2010 ^33^ Table 9.1.

**Figure 6.**
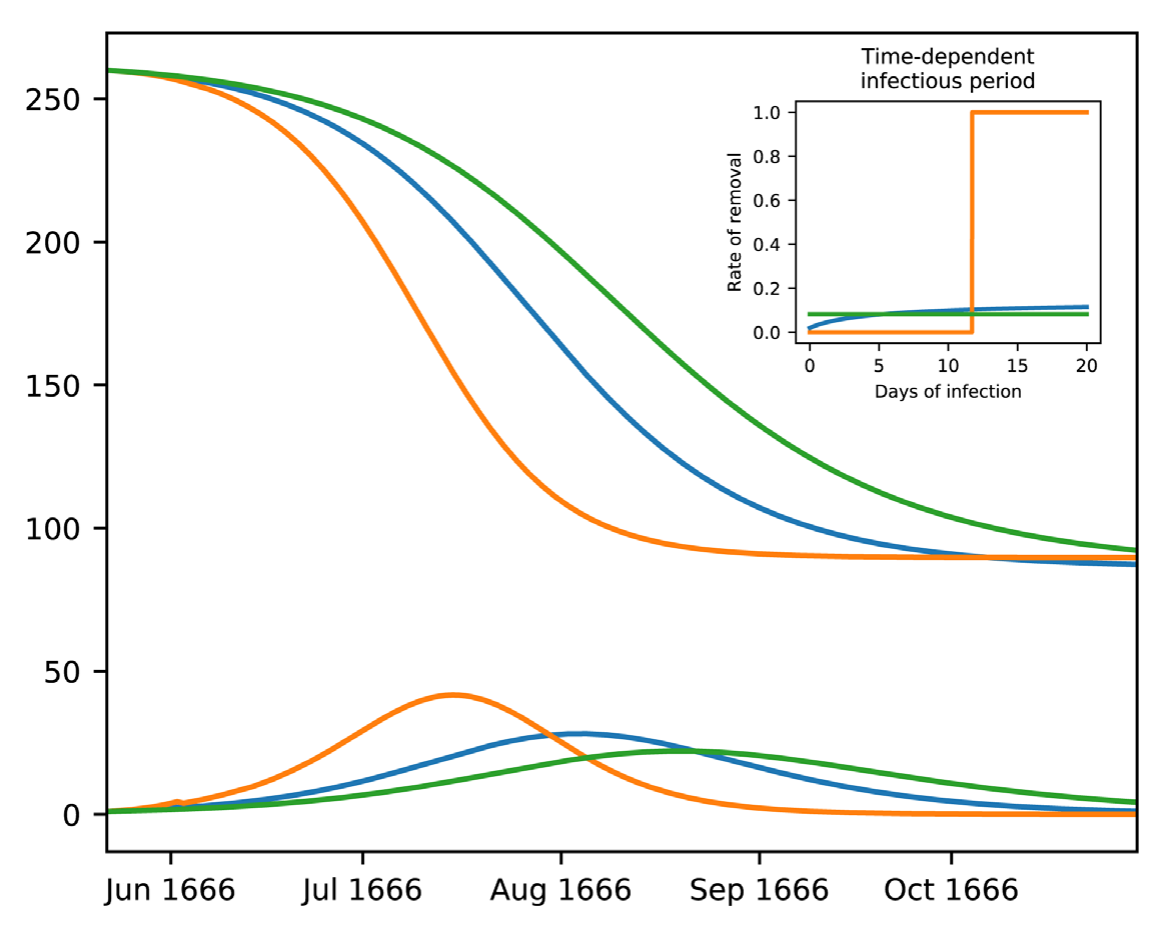
The effect of age-structure in modelling epidemics. The outbreak trajectory shown in Figure 5(b) (blue lines) is compared with alternative forms of mortality. Green lines indicate time-independence where the gamma distributed infectious period has *σ* = *µ*. Orange lines indicate that the infectious period has a precise length of *µ* = 11 *.*71 days ( *σ* = 0).

Figure 6 demonstrates the effect of age-structure on outbreak dynamics by comparing model output with different characteristics of the infectious period. The blue lines indicate the trajectory matching the Great Plague in Eyam. Please note that the corresponding infectious period is only slightly time-dependent where there is a minor difference between the mortalities of newly infected and long-time infectious individuals (plotted in the inset). If the rate of exit from the infectious stage is completely independent from the duration spent in the stage (as is the case with canonical SIR models), the outbreak duration increases (the green lines). In the opposite scenario, where the length of the infectious case is precise, the entire outbreak resolves rapidly as seen in the orange trajectory. Since time-dependence has a significant impact on outbreak trajectory, using realistic mathematical formulations to infer outbreak parameters, such as the incubation period and the rate of infection, becomes a critical step in developing predictive epidemic models.

## Summary

The sPop packages are designed to incorporate age dependence in discrete-time population dynamics models. The underlying model incorporates survival, development, and migration processes, and it can accommodate both deterministic and stochastic dynamics. In order to promote applications, three versions of the model were implemented: the R and Python implementations are aimed at educational and introductory level use, while the C implementation offers further optimisation and high-speed simulations. This paper demonstrated that the model is capable of representing age-structured population dynamics in different contexts including insect population dynamics, host-parasite/prey-predator interactions, and infectious disease epidemiology. Future research concerns optimising the implementation for faster simulations, implementing the model in continuous time domain for improved accuracy, and incorporating accumulative development processes under varying environmental conditions for a broader and more accurate representation of biological processes.

## Software and data availability

R implementation of sPop:

- Available from: https://cran.r-project.org/web/packages/albopictus/
- Source code: http://github.com/kerguler/albopictusR
- Archived source code as at time of publication: https://doi.org/10.5281/zenodo.1685054 ^34^
- License: GPLv3

C and Python implementation of sPop:

- Available from: https://pypi.org/project/albopictus/
- Source code: https://github.com/kerguler/albopictus
- Archived source code as at time of publication: https://doi.org/10.5281/zenodo.1685289 ^35^
- License: GPLv3

### Extended data

All data and source code for running the examples and plotting the figures in this manuscript are provided in the Extended data:

- test_spop.R: R script file for Section Implementation:R. https://doi.org/10.6084/m9.figshare.12957665 ^24^
- test_spop.py: Python script file for Section Implementation:Python. https://doi.org/10.6084/m9.figshare.12957710 ^25^
- test_spop.c: C code file for Section Implementation:C. https://doi.org/10.6084/m9.figshare.12957725 ^26^
- plot_test_spop.R: R script file for plotting Figure 1. https://doi.org/10.6084/m9.figshare.12957740 ^36^
- case_studies.py: Python script file for Section Use Cases. https://doi.org/10.6084/m9.figshare.12957734 ^28^
