# Supplementary material for: sPop: Age-structured discrete-time population dynamics model in C, Python, and R
Source: F1000Res. 2020 Sep 28;7:1220. Originally published 2018 Aug 8. [Version 3] doi: 10.12688/f1000research.15824.3 (PMC6198259; doi:10.12688/f1000research.15824.3)
Supplement: Peer review history

# Peer review: Reviewer response for version 1

**DOI:** 10.5256/f1000research.17272.r39332

**Published:** 2018-10-22

**Authors:** Matthew Silk

**Affiliations:** 1 Centre for Ecology and Conservation, University of Exeter, Penryn, UK

In this manuscript Erguler introduces the software tool that can be applied to modelling discrete time, age-structured population dynamics in R, Python and C. The basics of using the software in all three programming languages are well covered and the examples of its use and compelling and clearly explained. The code and equations provided seemed accurate and relevant (albeit with the caveat that I am an R user only, and an ecologist rather than mathematical biologist).

 I feel that a bit more background provided in the introduction would be beneficial, in particular an overview of the research areas where these models are used (as per the examples) and the reasons that age-structured models can be important to use.

 It would also be good to see some of the caveats/constraints of this sort of modelling framework discussed at some point so that a reader was better able to decide if it is a useful tool for them.

 Finally, there were a couple of places where I felt a bit more detail would be helpful:

- As someone who doesn't work on insects, a clarification of what constitutes a development cycle would be helpful.
- It would be helpful to more clearly explain the perturbate() function and perhaps provide an illustrative example of when it would be used.
- In the Nicholson's blowflies example it is not clear to me from the figure that relative heights of the minor cycles are "changing drastically". I feel this needs to be better depicted or explained.
- When introducing the plague model it would be helpful to explain how it is age-structured and clarify that this applies to the pathogen.

Otherwise I have a set of very minor changes/suggestions.

**Introduction:**

 Paragraph 1 - Suggest editing the first sentence to say that populations are heterogeneous, and one of the ways that they are heterogeneous is because age can cause differences among individuals. This feels like a clearer description to me.

 Paragraph 1 - Add "For example," to the start of the second sentence.

 Paragraph 1 - Suggest the end of the first paragraph needs some references to support this point.

 Paragraph 2 - Sentence starting "This approach" is difficult to read; perhaps split up to cover the deterministic/stochastic and discrete/continuous points separately?

**Implementation:**

 In the R section in paragraph starting "By defining..." - "THE same principles apply...."

 In the C section in paragraph starting "The macro..." - "unoptimised implementations of the NEGATIVE binomial"

**Use Cases:**

 Paragraph 1 - Change guideline to guidelines in final sentence.

 Age-structured host-parasite interactions - clarifying what Hastings was modelling originally would be helpful - title of reference says predators while in the text it says parasites?

 Paragraph starting "Hastings (1984)..." - change resembles to resemble.

 Eyam example - It was in 1665-1666 not in 1965-1966 as currently stated in the text.

**Summary:**

 First sentence of the summary reads awkwardly and could do with rephrasing.

 Figure 4: One of the points is only partially on each graph - it would be good to change this.

 Figure 4 legend: Misspelling of Eyam

 Overall this manuscript is clearly written and explained and introduces a potentially useful piece of software available to a wide range of programmers.

Are the conclusions about the tool and its performance adequately supported by the findings presented in the article?

Yes

Is the rationale for developing the new software tool clearly explained?

Partly

Is the description of the software tool technically sound?

Yes

Are sufficient details of the code, methods and analysis (if applicable) provided to allow replication of the software development and its use by others?

Yes

Is sufficient information provided to allow interpretation of the expected output datasets and any results generated using the tool?

Yes

Reviewer Expertise:

Disease ecology; Wildlife demography; Animal social structure and behaviour

I confirm that I have read this submission and believe that I have an appropriate level of expertise to confirm that it is of an acceptable scientific standard.

# Response

**Published:** 2018-11-29

**Authors:** Kamil Erguler

*I would like to thank Dr Silk for reviewing the manuscript and for his valuable comments and suggestions. I have submitted a revised version with an aim to address the issues raised in his review. My reply to the specific points raised can be seen below. The rest of his suggestions concerning minor corrections have been addressed in the manuscript accordingly.*

*Addressing specific issues raised in the review:*

- I feel that a bit more background provided in the introduction would be beneficial, in particular an overview of the research areas where these models are used (as per the examples) and the reasons that age-structured models can be important to use.
  Paragraph 1 - Suggest editing the first sentence to say that populations are heterogeneous, and one of the ways that they are heterogeneous is because age can cause differences among individuals. This feels like a clearer description to me.
  Paragraph 1 - Add "For example," to the start of the second sentence.
  Paragraph 1 - Suggest the end of the first paragraph needs some references to support this point.

*The revised version of the manuscript presents an extended introduction with additional links to the relevant literature and use cases. In addition, the definition of the model is revised and elaborated in the Methods section to convey in a more comprehensive manner the potential areas of use of the proposed modelling strategy. I believe the revised manuscript better describes the approach and places it in the right context.*

- It would also be good to see some of the caveats/constraints of this sort of modelling framework discussed at some point so that a reader was better able to decide if it is a useful tool for them.

*I added a section named Temporal resolution and accuracy to discuss the effect of time step size on the accuracy of numerical simulations. The section concludes with the assessment that the numerical error increases linearly with increasing step size. I believe that this will help readers to determine an appropriate step size for optimum accuracy given the availability of computational resources.*

- As someone who doesn't work on insects, a clarification of what constitutes a development cycle would be helpful.

*Development cycle is a concept introduced to the model to accommodate certain biological processes not necessarily related to insect development. In this revision, I used the analogy of human pregnancy to elaborate on the concepts of age (age of the mother), degree of development (duration of her pregnancy), and development cycle (the number of completed development processes which corresponds to the number of births the mother has given in this analogy). I believe that the analogy helps readers better conceptualise these processes and apply them to their work.*

- It would be helpful to more clearly explain the perturbate() function and perhaps provide an illustrative example of when it would be used.

*In this version of the manuscript, I renamed the method as "perturb", elaborated the discussion on its intended use, and provided a simple example to demonstrate how it can be used to model migration.*

- In the Nicholson's blowflies example it is not clear to me from the figure that relative heights of the minor cycles are "changing drastically". I feel this needs to be better depicted or explained. 

*I thank Dr Silk for pointing out this issue. In this version, I elaborated on the description of the variability in stochastic simulations and its likely correspondence to the observations of laboratory populations. I believe that readers will find this an improvement. *

- When introducing the plague model it would be helpful to explain how it is age-structured and clarify that this applies to the pathogen.

*In this version of the manuscript, I explained that the canonical SIR model is not age-structured; however, the underlying transmission dynamics might be better represented with an age-structured model. Especially in a case where disease duration or incubation period is of a certain length, the sPop packages are able to track each individual and trigger a state change only for the right ones and only when it is due. I believe that this revised version offers a clearer description of this capacity.*

- **Introduction:**
  Paragraph 2 - Sentence starting "This approach" is difficult to read; perhaps split up to cover the deterministic/stochastic and discrete/continuous points separately?

*Instead of various modelling frameworks, this version of the manuscripts presents examples of various contexts where intermittent stages have been used.*

- **Use Cases:**
  Age-structured host-parasite interactions - clarifying what Hastings was modelling originally would be helpful - title of reference says predators while in the text it says parasites?

*In this version of the manuscript, I stated that Hastings was modelling prey-predator interactions based on the host-parasite model, and presented the analogy between host-parasite and prey-predator interactions as considered by Hastings in his derivations.*

- **Summary:**
  First sentence of the summary reads awkwardly and could do with rephrasing.

*The sentence is rephrased.*

- **Figure 4:**
  One of the points is only partially on each graph - it would be good to change this.

*All Python-generated figures are re-plotted and *case_studies.py *is updated to improve the visibility of the data points.*

# Peer review: Reviewer response for version 1

**DOI:** 10.5256/f1000research.17272.r36953

**Published:** 2018-08-28

**Authors:** Juliane Liepe

**Affiliations:** 1 Max-Planck-Institute for Biophysical Chemistry, Göttingen, Germany

The manuscript “sPop: Age-structured discrete-time population dynamics model in C, Python, and R” by Kamil Erguler provides a comprehensive yet well summarised description of a novel software tool to model age-structured discrete-time population dynamics.

 The software tool is implemented in three different programming languages: R, Python and C. All three languages are most commonly used in scientific programming and therefore the presented tools are easily applicable and adaptable for a wide range of users. The manuscript highlights well the differences in implementation for the three programming languages. While R and Python implementations are easy to use and provide an excellent introduction to modellers new to age-structured population dynamics models, the C implementation provides computational speed, which is particularly relevant for parameter optimisation problems in this field.

 The manuscript guides the reader through three examples of the Python implementation. For each example, all necessary scripts are provided to reproduce the figures and the results described. While I have not had much experience with age-structured population dynamic models, I found it straight forward to follow the concept of the underlying model described in the manuscript.

 Overall this manuscript is well written and provides a hands-on guide to scientists to implement the discussed methods into their existing workflow.

Are the conclusions about the tool and its performance adequately supported by the findings presented in the article?

Yes

Is the rationale for developing the new software tool clearly explained?

Yes

Is the description of the software tool technically sound?

Yes

Are sufficient details of the code, methods and analysis (if applicable) provided to allow replication of the software development and its use by others?

Yes

Is sufficient information provided to allow interpretation of the expected output datasets and any results generated using the tool?

Yes

Reviewer Expertise:

NA

I confirm that I have read this submission and believe that I have an appropriate level of expertise to confirm that it is of an acceptable scientific standard.
